# Fondaparinux Outpatient Use for Patients with a Heparin-Induced Thrombocytopenia History: A Case Report and Review

**DOI:** 10.3390/pharmacy5010001

**Published:** 2016-12-22

**Authors:** Amy Christopher

**Affiliations:** Anticoagulation Clinic, St. Elizabeth Healthcare, 1 Medical Village Drive, Edgewood, KY 41017, USA; Amy.Christopher@stelizabeth.com

**Keywords:** anticoagulation, heparin-induced thrombocytopenia, factor-Xa inhibitors, novel oral anticoagulants, warfarin, periprocedural anticoagulation, subtherapeutic anticoagulation

## Abstract

The purpose of this article is to report a case of fondaparinux outpatient utilization for anticoagulation in a patient with a past medical history of heparin-induced thrombocytopenia (HIT) and discuss the options and need for future anticoagulation research in this unique patient population. A 63-year-old Caucasian female with a previous medical history of HIT thromboprophylaxed with warfarin for a pulmonary embolism presented to an anticoagulation clinic with a subtherapeutic international normalized ratio (INR) after missed warfarin doses. The patient was instructed to increase her warfarin dose and was prescribed fondaparinux daily injections until her INR was in range. The patient tolerated the fondaparinux therapy without thromboembolic, thrombocytopenia or bleeding occurrence. Fondaparinux therapy for HIT is controversial and differs between established guidelines. Currently, there is no studied use of fondaparinux for thromboprophylaxis in warfarin therapy outpatients with a HIT history who need thromboprophylaxis while undergoing therapy for a procedure, or those who have a subtherapeutic INR. Further study of the outpatient use of fondaparinux for this patient subset is needed to explore the potential benefit of an outpatient, less invasive, less expensive and potentially better tolerated option.

## 1. Introduction

The purpose of this case report and literature analysis is two-fold: to describe a patient case of fondaparinux outpatient utilization for a patient with subtherapeutic international normalized ratio (INR) and a history of heparin-induced thrombocytopenia (HIT) as well as inform health care providers of the clinical options of anticoagulants periprocedurally and for the thromboprophylaxis of warfarin-anticoagulated patients with a subtherapeutic INR complicated by a history of HIT.

HIT is the most frequent drug-induced, immune-mediated type of thrombocytopenia. It is associated with significant morbidity and mortality if unrecognized. Heparin-induced thrombocytopenia (HIT) occurs in up to 5% of patients who are exposed to unfractionated heparin (UFH) for 5 or more days. Due to the ability to monitor, recognize and discontinue heparin therapy, the mortality rate has decreased from 20% to 2%. Heparin-induced thrombocytopenia can be diagnosed via the enzyme-linked immunosorbent assay (ELISA) immunoassay or the serotonin-release assay (SRA) functional assay. The SRA functional assay remains the gold standard diagnostic test with 95% sensitivity and specificity.

However, the SRA functional assay is usually reserved for definite confirmation of antibody presence after receiving a positive result on an ELISA immunoassay test (HIT panel) [1,2].

Options for treatment of heparin-induced thrombocytopenia include argatroban or bivalirudin infusion. Argatroban and bivalirudin require hospital admission and dose adjustment for a therapeutic activated partial thromboplastin time (aPTT). There have been two small studies investigating the use of fondaparinux for treatment of HIT and it is mentioned as an option for HIT treatment by the American Society of Hematology (ASH) but not by the American College of Chest Physicians (ACCP). To the author’s knowledge, there is only one case series and no other retrospective reviews or studies involving outpatient use of fondaparinux for thromboprophylaxis in patients with a history of HIT [3,4].

Fondaparinux is a synthetic pentasaccharide factor Xa inhibitor given subcutaneously for thromboprophylaxis and treatment of venous thromboembolism. In contrast to heparin, fondaparinux does not inhibit thrombin and has no affinity to human platelet factor 4 (PF-4) antibody which is responsible for HIT. Fondaparinux does not bind to other plasma proteins and is renally excreted unchanged (up to 80%). The drug is contraindicated in patients with severe renal impairment (creatinine clearance less than 30 mL/min) and is not recommended for use in patients with a platelet count below 100,000 per cubic millimeter of whole blood. Steady-state plasma levels are typically reached after the third or fourth once-daily dose. Monitoring is usually not needed during therapy which offers an advantage over other anticoagulant options. Fondaparinux also has the advantage of using subcutaneously as an outpatient prescription and has potentially less risk of more serious side effects than the parenteral anticoagulants currently recommended.

Compared to low-molecular-weight heparin (LMWH), UFH has a 10-fold higher risk of developing HIT, whereas the pentasaccharide fondaparinux is rarely associated with HIT and has been described in only a few case reports. Depending on the source of UFH, the risk of HIT can increase or decrease with bovine UFH having a higher risk than porcine UFH. Fondaparinux has been reported to cause a similar clinical condition to HIT but ironically has been studied as a treatment alternative to argatroban and bivalirudin [2].

Fondaparinux has limited investigative reports for use in HIT patients. In a small retrospective review of hospital-admitted HIT suspected patients (*n* = 47), fondaparinux appeared to be similarly efficacious and safe in the prevention of new, recurrent or progressive thromboembolic events compared to direct thrombin inhibitors. Of the patients included in this study, only 12 of the patients were confirmed HIT panel/serotonin positive [1].

One recent case series of four patients with a past medical history of HIT utilizing fondaparinux for intra and perioperative anticoagulation concluded that case report results justified the off-label use of fondaparinux for peripheral vascular surgical interventions. Of note, only one of these case reports documented outpatient use of fondaparinux [3].

A 2015 propensity-matched study by Kang et al. demonstrated the similar effectiveness and safety of fondaparinux (*n* = 133) compared to argatroban and danaparoid in hospitalized patients with suspected HIT. A 2016 publication review of HIT commented about this study, stating, “although there are several possible limitations to this study, it certainly supports the need for further investigation into fondaparinux’s role in HIT treatment [5]”.

There are some differences between the ACCP and ASH guidelines for HIT therapy, including the utilization of fondaparinux. In the most recent ACCP guidelines, HIT therapy with fondaparinux was not recommended, because of a lack of non-case-report-based evidence. As previously mentioned, fondaparinux has been predicted to be a potential cause of HIT. Other more recent studies have shown effective results and minimal cross-reactivity with HIT serum, which would make it less likely to be a causative or precipitating agent [2].

ASH recommends the discontinuation of all heparin agents for patients with a HIT diagnosis or moderate to high suspicion of developing HIT. ASH treatment recommendations for HIT include argatroban, danaparoid, bivalirudin, and fondaparinux. Danaparoid is no longer available in the United States but is available in other countries. There is a statement in the ASH recommendations noting the difference from the ACCP recommendations: “other experts believe that fondaparinux is an important treatment option, especially in stable, non-critically ill patients”. With regards to monitoring of fondaparinux, the ASH guideline states that some experts recommend adjusting dose to peak anti-Xa activity of 1.5 fondparinux-specific units/ml. The ASH recommendations also state that some experts do not recommend routine monitoring during fondaparinux therapy [6].

There are also warfarin dosing complications while transitioning argatroban back to warfarin. Clinicians must recognize the cumulative and unpredictable effect of argatroban and warfarin on the INR lab test. The argatroban package insert recommends overlapping argatroban with warfarin therapy until INR is greater than 4. After the INR is greater than 4, clinicians should wait 4 to 6 h, stop argatroban then continue solely with warfarin therapy if the INR remains within the desired therapeutic range [2].

The direct oral anticoagulants such as dabigatran, rivaroxaban, and apixaban are also used off-label for the treatment of HIT. Dabigatran is an oral direct thrombin inhibitor while rivaroxaban, apixaban, edoxaban and betrixaban are oral factor Xa inhibitors. The predictable pharmacokinetic and pharmacodynamics effect of these agents make them easy to dose without the need of routine laboratory monitoring. Unlike heparin, none of the direct oral anticoagulants interact with PF4 due to their structure and therefore would not be involved in the development of HIT. Most experience is favorable but anecdotal in patients with variable laboratory confirmation of a diagnosis of HIT. One systematic review demonstrated 36 patients with suspected HIT (91% with positive antibodies) who were treated with dabigatran (*n* = 13), rivaroxaban (*n* = 18) or apixaban (*n* = 5) without bleeding or thrombosis during treatment. Rivaroxaban has the most literature regarding use in patients with confirmed or suspected HIT, including several case reports without thrombosis or bleeding complications. One prospective cohort study of 22 rivaroxaban-treated patients with suspected HIT resulted in only one progressive thrombosis. Currently, there is also a prospective multicenter clinical trial investigating the efficacy of rivaroxaban in the treatment of HIT. There are also case reports of the use of dabigatran (three reports) and apixaban (one report) in patients with suspected or confirmed HIT with favorable results. To the author’s knowledge, there are no case reports or studies reporting the utilization of edoxaban or betrixaban for the treatment of HIT [4,5,7].

There are also some significant differences in adverse events among the guideline-approved anticoagulant choices for HIT compared to fondaparinux as noted in Table 1 below. Argatroban use in patients undergoing cardiac and vascular surgery has been associated with bleeding requiring reoperation and even need for allogenic transfusions [3]. Fondaparinux has a much lower risk of hypotension, hypersensitivity reactions, gastrointestinal (GI) hemorrhage, chest pain, and minor bleeding compared to argatroban and bivalirudin.

The DOACs have bleeding adverse events higher than fondaparinux. Dabigatran adverse events include dyspepsia, gastric hemorrhage, erosive esophagitis, gastric ulcer (all >10%) and unspecified bleeding up to 16.5%. Rivaroxaban has a bleeding incidence greater than 10% with GI bleeding and anemia (including postoperative anemia) at an incidence of 1%–10%. Apixaban boasts less gastrointestinal bleeding but has a minor bleeding incidence of 11.7% and a 1%–10% incidence of postoperative anemia [7].

In the periprocedural and subtherapeutic INR patient, the most cost-effective anticoagulant therapy is not always a clear choice. When a warfarin anticoagulated patient has a subtherapeutic INR below 1.5 (or is off warfarin for a procedure) and is considered to be associated with a moderate to higher-risk of a venous thromboembolism, the CHEST guidelines recommend coverage with UFH or LMWH for at least 72 h and until the patient’s INR is in range. However, in a patient who has a history of HIT, the clinical conundrum forces us to ask: what should we do with patients that have a contraindication or an extreme precaution for the use of heparin?

Injectable factor X inhibitors like fondaparinux and novel or direct oral anticoagulants (NOACs or DOACs) have not been thoroughly studied in larger numbers for use in patients with HIT or a history of HIT needing periprocedural anticoagulation or additional anticoagulation due to subtherapeutic oral anticoagulant use.

## 2. Presentation of Case

KT is a 63-year-old Caucasian female thromboprophylaxed with warfarin for a pulmonary embolism history within the past 7 months as well as a lupus anticoagulant positive blood test at the time of the pulmonary embolism. She also has a past medical history of hypertension, hyperlipidemia, diabetes mellitus, depression, CAD, arthritis, heparin-induced thrombocytopenia (HIT) (positive serotonin-release unfractionated heparin assay) and anxiety. She presents to the anticoagulation clinic with a subtherapeutic INR of 1.4 after missing one or possibly two doses of warfarin in the past week.

KT was instructed to increase her warfarin dose and was given fondaparinux 7.5 mg subcutaneously once daily. The patient was also instructed to have a complete blood count done on day 3 and 5 of fondaparinux therapy. An INR recheck was scheduled after the fifth day of warfarin dose increase as well as fondaparinux daily use. The patient tolerated the fondaparinux therapy without any incidence of bleeding or thromboembolic event. The patient’s INR was 2.6 (within goal range) after five doses of increased warfarin dose and thromboprophylaxis coverage with daily fondaparinux. There was not a significant reduction in platelets during fondaparinux therapy. Laboratory values and medication list are noted in Table 2 and Table 3 below.

## 3. Discussion

The warfarin-anticoagulated patient with a subtherapeutic INR and a previous medical history of HIT in the present case report received and tolerated fondaparinux thromboprophylaxis as an outpatient therapy. Injectable factor X inhibitors like fondaparinux and novel or direct oral anticoagulants (NOACs or DOACs) have not been thoroughly studied in larger numbers for use in patients with HIT or a history of HIT needing periprocedural anticoagulation or additional anticoagulation due to subtherapeutic OAC use. The need for periprocedural or subtherapeutic INR anticoagulation coverage does occur occasionally in anticoagulated patients with a history of HIT, and further investigation is warranted due to the more costly options of argatroban and bivalirudin that also carry potentially more risk of serious adverse events.

Comparing the cost for argatrobapn and bivalirudin to fondaparinux, the five day treatment drug cost alone for the above patient is quite daunting: argatroban $1245 (starting dose of 2 mcg/kg/min) to $5727 (maximum dose of 10 mcg/kg/min) and bivalirudin $3435 versus fondaparinux $445 (five 7.5 mg daily sub Q syringes), not to mention the additional direct and indirect costs of a hospital admission with argatroban or bivalirudin intravenous treatment versus outpatient subcutaneous injections. The prices for a five-day therapy of apixaban, dabigatran, edoxaban, and rivaroxaban would be $58, $56.40, $47.20, and $58.01, respectively. Further study of safety and efficacy in this type of patient population with a history of HIT would provide substantial cost savings as well as the convenience of oral administration [8].

There also is a decrease in more serious adverse events related to the less invasive administration of subcutaneous fondaparinux versus parenteral administration of argatroban and bivalirudin, as evidenced by Table 1. There is significantly less risk of minor bleeding, chest pain and gastrointestinal hemorrhage with fondaparinux use compared to argatroban or bivlairudin. The patient in the above case report tolerated the fondaparinux well without symptoms of thromboembolism, bleeding/bruising symptoms or thrombocytopenia.

Another disadvantage of utilizing argatroban while transitioning to or from warfarin is the unpredictable effect argatroban has on the INR. Transitions to oral anticoagulation from argatroban should result in consultation of the health care institution where the patient will be treated to decide the potential effect on INR. Some experts recommend the alternative option of transitioning from argatroban to fondaparinux prior to the final transition to warfarin due to the lack of effect on INR. Therefore, the use of fondaparinux in place of a step down therapy from argatroban would be recommended to be studied in the future.

In the case of a patient with a HIT history and renal failure or thrombocytopenia, the investigation of fondaparinux or DOACs in this setting would be helpful due to the lack of options for anticoagulation.

When fondaparinux is used either off-label or in future studies in patients with a history of HIT, complete blood counts every 2–3 days and up to 5 days after discontinuation should be considered to detect any potential issues with thrombocytopenia.

## 4. Conclusions

Further studies with larger patient numbers are warranted for the outpatient use of fondaparinux or DOACs in the periprocedural and subtherapeutic INR setting for patients on warfarin therapy. If the outpatient use of fondaparinux were to be found to be a safe and effective thromboprophylactic therapy in this unique patient population, it could potentially avoid a hospital admission therapy with argatroban or bivalirudin that potentially could have more serious adverse events.

## Figures and Tables

**Table 1:** Adverse effects of Anticoagulants Used for heparin-induced thrombocytopenia (HIT).

Argatroban Adverse Effects	Bivalirudin Adverse Effects	Fondaparinux Adverse Effects
Hypersensivity reaction 13%	Hypotension 12%	Edema 8.7%
Hypotension 7.2%–10%	Nausea 15%	Rash 7.5%
Chest pain 15.2%	Backache 42%	Nausea/Vomiting/Constipation 6%–11%
GI hemorrhage 14.4%	Minor Bleeding 13.6%	Fever 13.6%
Sepsis 6%	Headache 12%	Anemia 19.6%
	Pain 15%	

**Table 2:** Pertinent Lab Values.

**Labs and Comments**	**Reference Range**	**21 January 2016 11:55**	**21 January 2016 23:00**	**4 February 2016 16:40**	**6 February 2016 06:04**	**19 September 2016 13:22**	**4 October 2016 07:36**
Platelets	144–423 × 10^3^ per cubic millimeter of whole blood	260	109 (L)	150	55 (L)	194	167
Comments		Heparin initiation at 13:51 during hospital admission		Heparin initiation bolus at 19:27 and infusion at 20:51	HIT suspected, heparin discontinued and labs below ordered during hospital admission	Pre-fondaparinux outpatient exposure	3 days post-fondaparinux outpatient exposure
**Lab Test**	**Reference Range**	**6 February 2016 13:39**					
SRA UF (Porcine)	Negative	Positive (A)					
SRA UF HEP LOW DOSE	Units: %	100					
Hep-Ind Thrombocytopenia PF4 Ab, IgG	≤0.399 OD	2.790 (H)					

**Table 3:** Medication List Prior to Outpatient Fondaparinux Use.

Current Daily Medications	
aspirin 81 mg	nitroglycerin 0.4 mg as needed
escitalopram 10 mg	pantoprazole 40 mg
furosemide 20 mg	sacubitril-valsartan 49/51 mg twice daily
metformin XR 750 mg	warfarin 1 mg tablets
metoprolol succinate 50 mg	warfarin 3 mg tablets
